# SideCow-VSS: A Video Semantic Segmentation Dataset and Benchmark for Intelligent Monitoring of Dairy Cows Health in Smart Ranch Environments

**DOI:** 10.3390/vetsci12111104

**Published:** 2025-11-19

**Authors:** Lei Yao, Jin Liu, Weinan Hong, Fanrong Kong, Zipei Fan, Lin Lei, Xinwei Li

**Affiliations:** 1 College of Artificial Intelligence, Jilin University, Changchun 130012, China; yaolei25@mails.jlu.edu.cn (L.Y.); hongwn24@mails.jlu.edu.cn (W.H.); 2 College of Software, Jilin University, Changchun 130015, China; liujin0623@mails.jlu.edu.cn; 3 College of Veterinary Medicine, Jilin University, Changchun 130062, China; kfr1224@163.com (F.K.); lixinwei100@126.com (X.L.)

**Keywords:** dairy cows, semantic segmentation, precision livestock farming, deep learning, disease diagnosis, computer vision, preventive strategies

## Abstract

Accurate and non-invasive monitoring of dairy cows is a cornerstone of precision livestock farming, paving the way for proactive health management and earlier disease detection. The development of robust, AI-driven diagnostic tools, however, is hindered by a dual challenge: scarce realistic video datasets and a lack of standardized benchmarks for deep learning models. To confront these issues, this study puts forward SideCow-VSS, a video semantic segmentation dataset comprising 921 side-view clips with dense, pixel-level annotations of dairy cows under variable on-farm conditions. We systematically evaluated eight deep learning architectures, from classic convolutional neural networks to state-of-the-art Transformers. The evaluation highlighted a clear performance trade-off: the Mask2Former model with a Swin-L backbone yielded the highest mIoU at 97.32%, making it well-suited for detailed morphological analysis. In contrast, the lightweight PIDNet-s model achieved the fastest inference speed of 59.5 FPS, demonstrating its potential for real-time behavioral alerting systems. This work delivers a foundational resource and quantitative framework to inform model selection, accelerating the creation of computer vision systems for automated health monitoring and adopting preventive strategies against key metabolic and immunological disorders in dairy production.

## Simple Summary

Automated monitoring of dairy cow health is a key goal of modern smart farming. While technologies like wearable sensors exist, computer vision offers a powerful, non-invasive alternative using cameras and AI to analyze animal appearance and behavior. However, developing reliable vision-based AI is challenged by the lack of high-quality video data from real farms. To address this, we created the SideCow-VSS dataset, a collection of 921 precisely annotated, side-view video clips. This perspective is valuable for assessing health indicators like body condition and gait. We then tested eight AI models on this dataset, revealing a clear trade-off between accuracy and speed. Our results show some models are ideal for detailed analysis, while others are fast enough for real-time farm alerts. This study provides a public resource and a practical guide for creating next-generation automated health monitoring systems for dairy cattle.

## 1. Introduction

Effectively managing individual animal health remains one of the most pressing challenges in modern dairy production, with direct effects on animal welfare, productivity, and the long-term economic sustainability of farms [1,2,3,4,5,6]. The ability to detect early signs of health disorders, such as those related to metabolic stress or lameness, is crucial to prevent irreversible damage and economic loss [7]. In recent years, the field has experienced a paradigm shift from traditional, subjective observation toward data-driven Precision Livestock Farming (PLF). By integrating artificial intelligence (AI) and computer vision technologies, PLF enables continuous, automated, and objective health monitoring, offering veterinarians and farm managers powerful tools for early diagnosis and disease prevention [8,9,10].

At the core of these intelligent systems lies the ability to recognize subtle visual biomarkers that often precede the onset of clinical disease. For instance, automated Body Condition Scoring (BCS) has become a vital approach for assessing the energy balance and nutritional status of cows [11,12,13], while deviations in posture or gait may serve as early indicators of compromised welfare [14,15,16]. While some approaches rely on wearable sensors like accelerometers [14], vision-based systems offer a non-invasive alternative for gait analysis [16]. Nevertheless, the success of all such visual monitoring applications depends critically on one foundational step: accurately and reliably segmenting each animal from the complex, dynamically changing background typical of real farm environments.

To address this fundamental challenge, computer vision research within PLF has advanced rapidly. Early studies concentrated on static tasks such as quantifying feed intake using RGB-D cameras [17] or detecting dairy cows via customized YOLO-based models like YOLOv5-ASFF, building upon foundational object detection frameworks [4,18,19,20]. For animal identification, research diverged into two principal directions: non-invasive biometric recognition based on natural body features, and attachment-based systems that identify artificial markers such as ear tags through a combination of object detection and optical character recognition (OCR) [21]. Over time, the research focus shifted from static detection toward dynamic multi-object tracking (MOT) with algorithms like YOLO-BYTE, enabling consistent identity tracking even in crowded barns—a prerequisite for longitudinal behavioral analysis [22,23,24,25,26]. This evolution opened the door to more sophisticated analytical frameworks. For example, lameness detection has evolved into a multi-stage process involving animal detection, keypoint-based pose estimation, and biomechanical classification to quantify gait asymmetries [14,15]. More recently, integrated diagnostic systems have begun to fuse vision data with complementary modalities such as wearable sensors and genomic information, uncovering complex metabolic and immune disorders and signaling a transition from isolated analytical tools to holistic AI-driven health monitoring ecosystems [27].

The progress of these applications, of course, is tightly coupled with the availability of high-quality, annotated datasets. Although large-scale general-purpose datasets such as COCO and ImageNet have been widely used for model pre-training [28,29,30], the domain gap between these sources and real farm environments—characterized by uneven lighting, occlusions, and non-standard animal postures—has driven the development of livestock-specific data resources [31]. Public repositories like Kaggle and Roboflow have begun to host dedicated dairy cows segmentation datasets, and large-scale multimodal projects such as MmCows now provide synchronized RGB, depth, and sensor data for advanced livestock research [32]. While these resources are valuable for static image analysis or multimodal research, a direct comparison reveals a persistent gap in resources designed for dynamic, video-based semantic segmentation. Despite these initiatives, a crucial gap persists: the lack of a publicly accessible, video-based dataset with dense, pixel-level semantic annotations that capture the temporal dynamics of dairy cows in authentic barn environments. Such a dataset is indispensable for training and evaluating models capable of operating robustly in practical disease-monitoring contexts.

Meanwhile, deep learning architectures for semantic segmentation have advanced at an equally rapid pace. The field has moved from foundational Convolutional Neural Networks (CNNs) such as U-Net [33] and DeepLabV3+ [34] toward modern Transformer-based architectures. Recent innovations like SegFormer, SegNeXt, and Mask2Former have redefined the state of the art by combining hierarchical attention mechanisms with efficient decoding strategies [35,36,37]. Despite their strong performance on general benchmarks, a comprehensive evaluation is needed to understand their potential and address the trade-offs between segmentation accuracy and computational efficiency within the specific context of dairy cows health monitoring.

This dual challenge—the absence of a suitable video-based dataset and the lack of a systematic model benchmark—creates a significant bottleneck for progress in veterinary-oriented perception research. To overcome these barriers, we make two key contributions. First, we introduce SideCow-VSS (Side-View Video Semantic Segmentation Dataset), a new video-based dataset designed specifically for intelligent monitoring of dairy cows. It contains 921 five-second side-view clips (46,050 frames) with dense, pixel-level annotations captured under real farm conditions. The dataset emphasizes side-view perspectives, which are particularly informative for health evaluations such as BCS and lameness assessment. Second, we present a comprehensive benchmark of eight representative deep learning architectures, spanning from classical CNNs and lightweight real-time networks to cutting-edge Transformer models. Our evaluation culminates in a detailed “precision versus speed” analysis, providing actionable insights for selecting the optimal architecture for a given application—whether for high-accuracy offline diagnostics or real-time on-farm disease surveillance.

Together, these contributions establish a foundational resource and a quantitative framework intended to fundamentally improve how AI models perceive and understand dairy cattle in visual data. By providing a robust solution to the prerequisite challenge of segmentation, our work serves as a critical enabler for the development of more accurate and reliable downstream applications, including automated health assessment, early disease detection, and precision management in dairy farming.

## 2. Materials and Methods

### 2.1. Dataset Construction

To ground our study in a realistic setting, we developed a new video dataset, hereafter referred to as SideCow-VSS, specifically to address the need for a dynamic benchmark in dairy cows segmentation. The raw footage was gathered over five consecutive days at a commercial dairy farm in Changchun, China, which housed a herd of approximately 500 Holstein-Friesian dairy cows in a typical free-stall barn system where cows had free access to cubicles and a feeding alley. To capture walking sequences, data was collected using a commercial 2 K surveillance Sinlihe camera, Shenzhen, China specifically positioned to monitor a lane leading to the milking parlor. The camera was mounted on a side wall at a height of 3 m, providing a clear side-view perspective of the animals as they passed, and recorded video at a resolution of 2560 × 1920 pixels and 15 FPS. Over the five-day collection period, this process generated a raw dataset of over one million frames ( 1 M total frames). Since our data collection was entirely observational and non-invasive, involving no alteration to animal management, specific ethical approval was not required. For the practical purpose of annotation, the continuous footage was pre-processed by downsampling it to 10 FPS and filtering for non-blurry, informative sequences, which resulted in the final 46,050 frames (approx. 46 k) selected for annotation.

**Figure 1:**
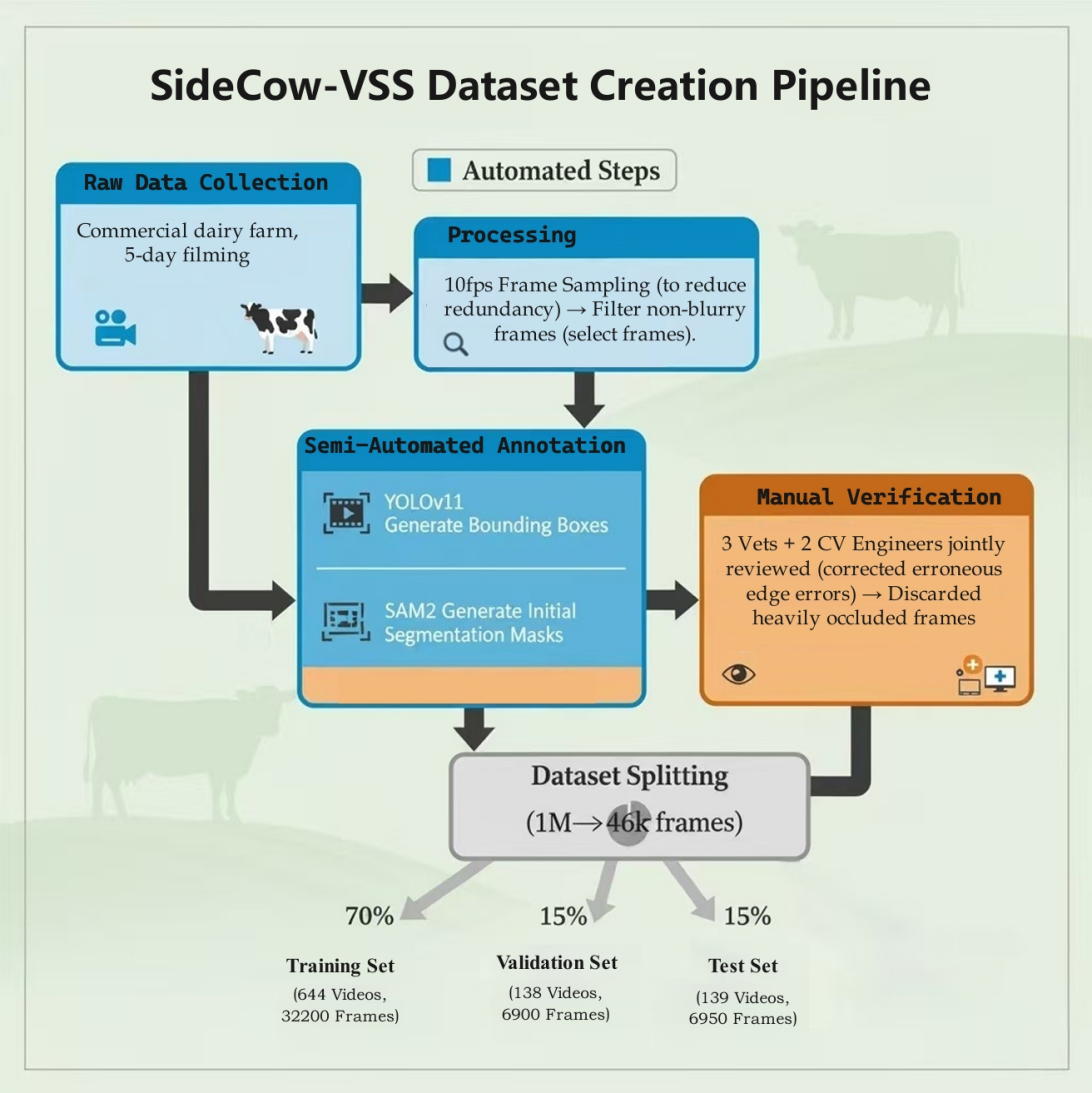
The creation pipeline of the SideCow-VSS dataset. The process begins with raw data collection from a commercial dairy farm. Automated steps include frame sampling and semi-automated annotation using YOLOv11 for bounding box generation and SAM2 for initial mask creation. A crucial manual verification step, conducted by veterinarians and computer vision engineers, ensures the quality and accuracy of the final annotations before the dataset is partitioned into training, validation, and test sets.

Given the sheer volume of frames, it became clear that a purely manual annotation process would be infeasible. We therefore designed an efficient semi-automated pipeline that followed a two-stage process. In the first stage, we used a YOLOv11 object detection model [38] to generate initial bounding box proposals for the cattle in each frame. These bounding boxes then served as prompts for the second stage, where SAM2 [3] produced precise, initial segmentation masks for each animal. It is important to note that the YOLOv11 component served only as an internal proposal generator to guide the SAM2 model; its performance was not formally evaluated, as the final accuracy of all annotations was determined by the subsequent manual verification step. The entire semi-automated pipeline is illustrated in Figure 1.

Of course, no automated annotation is perfect, so a meticulous manual verification and curation step was indispensable for ensuring the quality and accuracy of the final dataset. This human-in-the-loop approach involved trained annotators who inspected every generated mask, carefully refining the boundaries to match the true contour of the cow. Any frames containing significant segmentation errors or where the animal was severely occluded (e.g., >50% of the body obscured) were discarded from the final pool. The result of this rigorous curation process was the final dataset, consisting of 921 continuous 50-frame (five-second) video sequences suitable for robust model training and evaluation.

For standardized model development and comparison, the final Side-VSS dataset was partitioned into training, validation, and test sets. The data was organized as follows: the training set is composed of videos 001 to 644 (representing 70% of the data), the validation set contains videos 645 to 782 (15%), and the test set includes the remaining videos from 783 to 921 (15%). This video-ID-based split ensures that all frames from any given 5-s sequence are contained entirely within a single set (training, validation, or test), thereby strictly preventing any data leakage between the splits and ensuring a fair evaluation of model generalization.

### 2.2. Benchmark Models

To establish a comprehensive and informative benchmark, we selected a diverse set of eight semantic segmentation architectures. Our selection spans from classic CNN based models to cutting-edge Transformer approaches, offering a holistic view of the current technological landscape. The models were carefully chosen to represent different design philosophies and to examine the trade-off between segmentation accuracy and inference speed.

To anchor our benchmark in established baselines, we included two widely adopted CNN-based models. U-Net [33] was selected for its iconic encoder-decoder architecture, which has demonstrated remarkable performance, particularly in biomedical image segmentation. We also included DeepLabV3+ [34], a strong and well-recognized benchmark model known for its effective use of atrous convolutions and powerful backbones such as ResNet to capture multi-scale contextual information [39].

Recognizing that many on-farm applications require real-time operation, we also incorporated models specifically designed for high efficiency. The PIDNet family [40] was chosen for this purpose. To investigate the performance trade-offs within this architecture, we evaluated both its smallest variant, PIDNet-s, and its largest variant, PIDNet-l.

To evaluate the latest advancements in deep learning-based segmentation, we benchmarked several state-of-the-art Transformer architectures. The SegFormer series [35], known for its simple yet powerful hierarchical design, was included with both its smallest, SegFormer-b0, and largest, SegFormer-b5, variants to analyze the impact of model scaling. We further incorporated SegNeXt-L [36], a recent high-performing model that enhances context aggregation through multi-branch attention mechanisms. Finally, to explore the upper limits of segmentation accuracy, we integrated Mask2Former [37] with a Swin-L backbone [41]. This model represents the current frontier of Transformer-based segmentation, combining a hierarchical backbone pre-trained on the extensive ImageNet-22K dataset with a mask-attention head capable of highly precise and boundary-aware segmentation, drawing from advances in universal segmentation tasks [42].

This diverse set of models provides a robust and balanced foundation for comparative analysis, enabling a nuanced understanding of how architectural choices affect performance in the context of real-world dairy cows segmentation.

### 2.3. Implementation Details and Evaluation Metrics

To ensure a fair and reproducible comparison, all models were benchmarked under a unified experimental framework. All experiments were conducted on a single workstation equipped with an Intel Core i9-14900K CPU, 128 GB of RAM, and an NVIDIA GeForce RTX 4090 GPU, using a software environment based on PyTorch 2.1.0, CUDA 12.1, and MMEngine 0.10.7. For result stability, a fixed random seed of 42 was used throughout all training runs, and the cudnn_benchmark flag was enabled to optimize computational performance.

**Figure 2:**
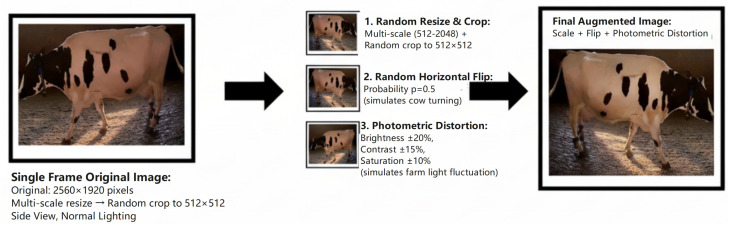
Visualization of the data augmentation pipeline applied during model training. An original image from the dataset undergoes a series of transformations, including (1) random scaling, (2) random horizontal flipping to simulate different viewing angles, and (3) photometric distortion to simulate variations in farm lighting conditions. These techniques artificially expand the training data to enhance model robustness and generalization.

During training, all images were processed through a consistent data augmentation pipeline that included random scaling, random cropping to a fixed size of 512×512 pixels, horizontal flipping with a probability of 0.5, and photometric distortions. These transformations are visually demonstrated in Figure 2. For validation and testing, images were resized to a short side of 512 pixels while preserving their aspect ratio, and inference was performed on the full-resolution images.

Each model was trained for 40,000 iterations using an iteration-based training loop. Model performance was evaluated on the validation set every 4000 iterations, and the checkpoint achieving the highest mean Intersection over Union (mIoU) on the validation set was retained for final testing. While most models followed a polynomial (Poly) learning-rate decay schedule, the choice of optimizer and other hyperparameters varied depending on the model architecture. Detailed configurations for each model are provided in Table A1 in the Appendix A.

The performance of all models was quantitatively assessed using a standard suite of semantic segmentation metrics, including mean Intersection over Union (mIoU), mean Dice Coefficient (mDice), overall Accuracy (aAcc), mean Accuracy (mAcc), mean F-score (mFscore), mean Precision (mPrecision), and mean Recall (mRecall). Among these, mIoU was chosen as the primary indicator for model comparison and selection, as it remains the most widely accepted and discriminative metric in semantic segmentation research.

## 3. Results

The evaluation results of the eight benchmarked model architectures on the SideCow-VSS test set are presented from quantitative, comparative, and qualitative perspectives. This approach provides a holistic understanding of each model’s performance in a realistic farm environment.

### 3.1. Quantitative Comparison

The primary quantitative results of our benchmark are summarized in Table 1. This table details the performance of each model, focusing on the mean Intersection over Union (mIoU) for segmentation accuracy and Frames Per Second (FPS) for inference speed, which includes data pre-processing time.

As shown in the table, a clear performance hierarchy emerged. The Transformer-based Mask2Former with a Swin-L backbone set the upper bound for accuracy, achieving the highest mIoU of 97.32%. Following closely was SegNeXt-L, another advanced model, which scored an mIoU of 97.00%. At the other end of the spectrum, the lightweight PIDNet-s model demonstrated the highest inference speed, reaching 59.5 FPS, making it a strong candidate for real-time applications. Notably, the classic CNN-based models, U-Net and DeepLabV3+, delivered robust and highly competitive accuracy (96.97% and 96.96% mIoU, respectively), demonstrating their continued relevance as strong baselines.

### 3.2. Accuracy Versus Speed Trade-Off

**Figure 3:**
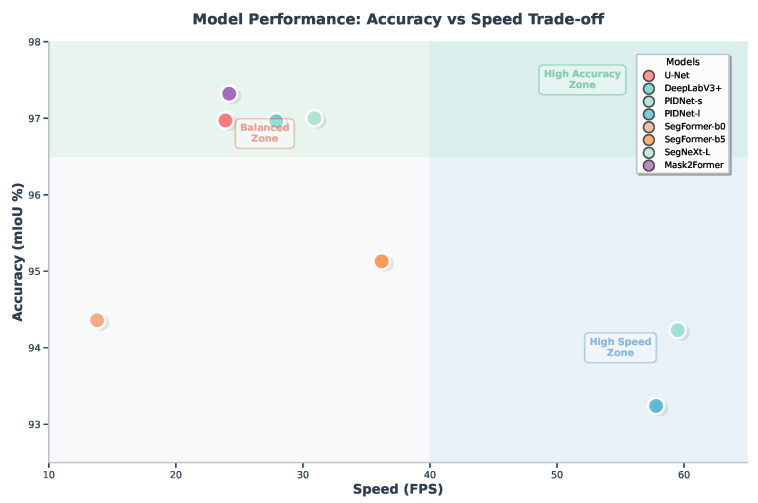
Accuracy (mIoU) versus speed (FPS) trade-off for the eight benchmarked models on the SideCow-VSS test set. The plot is divided into three conceptual zones: a high-accuracy zone (**top-left**), a high-speed zone (**bottom-right**), and a balanced zone. The ideal model would be located in the top-right corner. This visualization highlights the distinct performance profiles of different architectural families.

To visually illustrate the critical trade-off between segmentation accuracy and inference speed, the performance data from Table 1 is plotted in Figure 3. In this plot, the vertical axis represents accuracy (mIoU), while the horizontal axis represents speed (FPS). An ideal model would be situated in the top-right corner, signifying both high accuracy and high speed.

The plot reveals several distinct performance clusters. Mask2Former and SegNeXt-L occupy the high-accuracy, moderate-speed quadrant, achieving over 97% mIoU at speeds between 24 and 31 FPS. Conversely, the PIDNet family resides in the high-speed region on the far right; PIDNet-s, in particular, stands out as the fastest model by a significant margin at 59.5 FPS, albeit with a lower mIoU of 94.23%. Models such as DeepLabV3+ and SegFormer-b5 strike a balance between the two extremes, offering respectable accuracy above 95% with practical inference speeds. This visualization provides an intuitive roadmap for selecting an appropriate model based on specific application priorities.

### 3.3. Qualitative Analysis

**Figure 4:**
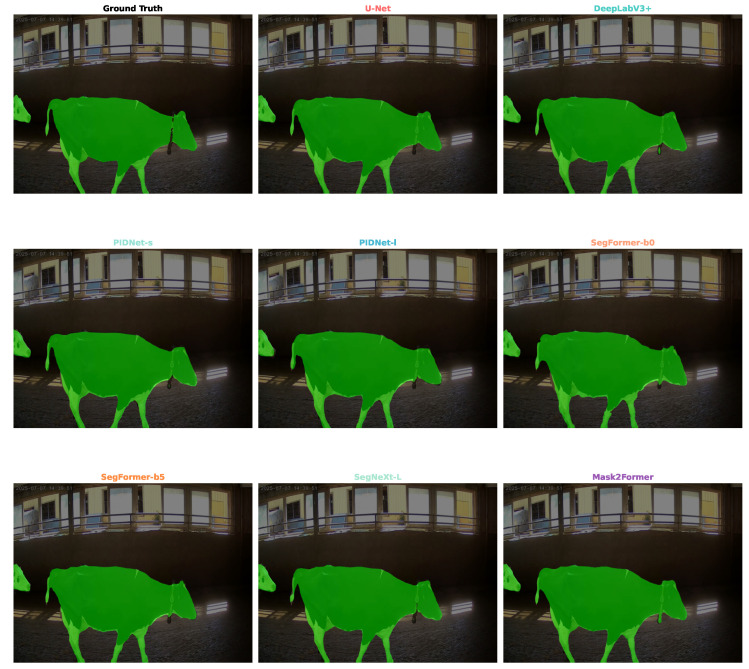
Qualitative comparison of segmentation results from representative models on a challenging example from the test set. While all models capture the general shape, Mask2Former provides the most precise boundary delineation, especially around the legs and tail, closely matching the ground truth. In contrast, models like PIDNet-s and SegFormer-b0 may produce slightly less accurate contours, illustrating the trade-off between speed and fine-grained accuracy.

To complement the quantitative metrics, visual inspection of segmentation results on challenging frames highlights the practical differences between the architectural paradigms (Figure 4).

While all models effectively segment the main body of the cow under simple conditions, their performance diverges in complex scenarios. The state-of-the-art model, Mask2Former, excels in delineating fine-grained details, such as the contours of the legs and tail, and shows greater robustness to occlusions and varied lighting. In contrast, the real-time model, PIDNet-s, while generally effective, occasionally struggles with intricate boundaries or areas with subtle texture changes. The classic DeepLabV3+ model provides a solid baseline but can produce less precise edges compared to its Transformer-based counterparts. These visual results corroborate the quantitative findings in Table 1 and underscore the tangible differences in segmentation quality among the models.

## 4. Discussion

Our systematic benchmark of eight semantic segmentation models on the SideCow-VSS dataset provides more than just a technical comparison; it offers a foundational roadmap for enhancing the visual perception capabilities of AI in dairy farming. A central theme emerging from our results is the persistent trade-off between segmentation accuracy—which can be interpreted as the depth of visual understanding—and computational speed. While modern Transformer-based architectures have pushed the boundaries of precision, this performance comes at a cost, creating a clear decision point for real-world deployment. This “precision versus speed” dilemma, visually captured in Figure 3, is not merely a technical footnote; it reflects the diverse operational needs for different levels of automated animal health monitoring.

This finding has profound implications for tailoring technological solutions to specific agricultural challenges. For applications where analytical depth is paramount, such as in genetic research based on detailed morphology, accuracy is non-negotiable. Similarly, while some of the most comprehensive Body Condition Scoring (BCS) systems have utilized 3D data, recent studies continue to demonstrate the significant potential of using 2D side-view images to extract crucial morphological features for BCS assessment [43,44]. In such 2D-based approaches, the precision of the initial body contour segmentation—as provided by models like Mask2Former (97.32% mIoU)—is a critical first step that directly impacts the accuracy of the final score. On the other hand, for applications demanding immediate intervention, such as in real-time early-warning systems for lameness or calving, inference speed becomes the critical bottleneck. In these contexts, a model like PIDNet-s, with its remarkable 59.5 FPS, emerges as the more practical option. Our benchmark, therefore, serves as the first quantitative framework in this domain to empower stakeholders to make an informed choice, aligning the computational cost of a model with the specific analytical detail their application requires.

The observed performance gap between model families can likely be attributed to fundamental differences in their architectural designs. The Swin Transformer backbone in our top-performing model, for example, leverages hierarchical attention mechanisms that are adept at capturing multi-scale features—a crucial capability when dealing with variations in dairy cattle size and camera distance. Furthermore, the innovative mask attention mechanism in the Mask2Former head facilitates a more refined, object-aware segmentation compared to the more rigid convolutional kernels of traditional CNNs [45]. These architectural advantages translate into tangible qualitative differences, as illustrated in Figure 4, where the Transformer-based models consistently demonstrate more precise boundary delineation, particularly around challenging areas like legs and tails.

Finally, a candid acknowledgment of this study’s limitations is essential for contextualizing our findings and guiding future work. Our dataset, while robust, was collected from a single farm, meaning that model performance could vary with different cattle breeds, housing systems, or lighting conditions. Furthermore, our focus on semantic segmentation means the current models cannot distinguish between individual animals in close contact. Looking ahead, future research should prioritize several key avenues. First, expanding the dataset to encompass greater environmental diversity is crucial for enhancing model generalization. Second, extending this benchmark to instance segmentation models is a critical next step for enabling the individual animal tracking required for personalized health management. Lastly, the high-quality 2D segmentations established by this work can serve as a foundational component for more advanced phenotyping techniques, potentially including multi-view 3D reconstruction. Developing methods that can leverage precise 2D contours to infer 3D shape information represents an exciting frontier for creating even more accurate and robust health monitoring systems in the future.

## 5. Conclusions

In this paper, we addressed the challenge of robust semantic segmentation for dairy cows within a specific, complex farm environment, a foundational step for developing intelligent animal health and behavior monitoring systems. We introduced SideCow-VSS, a new video-based dataset, and conducted a comprehensive benchmark of eight deep learning models. Our findings reveal a distinct trade-off between accuracy and speed, with the Mask2Former model achieving the highest segmentation precision (mIoU of 97.32%), and the PIDNet-s model offering the fastest inference speed (59.5 FPS). The primary contribution of this work is a practical, data-driven framework that provides initial guidance for researchers and developers in selecting potentially suitable model architectures for specific veterinary and farm management applications. It is important to note that this study provides a foundational resource for the development of such applications, rather than an end-to-end health monitoring tool itself. This study lays the groundwork for future research, most notably the need to extend this benchmark to more diverse environments to validate the generalizability of these findings, as well as integrating temporal analysis and instance segmentation to enable personalized health management.

## Figures and Tables

**Table 1:** Performance comparison of semantic segmentation models on the SideCow-VSS test set. Speed is reported in frames per second (FPS), calculated based on the total inference time per frame, including pre-processing. The best results for accuracy (mIoU) and speed (FPS) are highlighted in bold.

Model	Backbone	mIoU (%)	Speed (FPS)
U-Net	-	96.97	23.9
DeepLabV3+	ResNet-101	96.96	27.9
PIDNet-s	-	94.23	**59.5**
PIDNet-l	-	93.24	57.8
SegFormer-b0	MiT-b0	94.36	13.8
SegFormer-b5	MiT-b5	95.13	36.2
SegNeXt-L	MSCAN-L	97.00	30.9
Mask2Former	Swin-L	**97.32**	24.2

## Data Availability

The raw data supporting the conclusions of this article will be made available by the authors on request.

## Abbreviations

AI	Artificial Intelligence
BCS	Body Condition Scoring
CNN	Convolutional Neural Network
FPS	Frames Per Second
mDice	mean Dice Coefficient
mIoU	mean Intersection over Union
MOT	Multi-Object Tracking
PLF	Precision Livestock Farming
SAM2	Segment Anything Model 2
SOTA	State-of-the-Art

## Appendix A. Model-Specific Hyperparameters

To ensure the reproducibility of our benchmark, the model-specific hyperparameters used for training are detailed in Table A1. While common settings such as the 40,000 training iterations and the 512 × 512 crop size were shared, key parameters like the optimizer, learning rate, and batch size were configured individually to suit each model’s architecture and memory requirements.

**Table A1:** Model-specific hyperparameters used in the experiments. All models were trained for 40,000 iterations.

Model	Backbone	Batch	Optimizer	Base LR	W-Decay	LR Sched.	Notes
U-Net	—	4	SGD	$1.0\times 10^{-2}$	$5.0\times 10^{-4}$	Poly	Standard Augmentation
DeepLabV3+	ResNet-101	4	SGD	$1.0\times 10^{-2}$	$5.0\times 10^{-4}$	Poly	SyncBN enabled
PIDNet-s	—	4	SGD	$1.0\times 10^{-2}$	$5.0\times 10^{-4}$	Poly	—
PIDNet-l	—	2	SGD	$1.0\times 10^{-2}$	$5.0\times 10^{-4}$	Poly	—
SegFormer-b0	MiT-b0	4	SGD	$1.0\times 10^{-2}$	$5.0\times 10^{-4}$	Poly	—
SegFormer-b5	MiT-b5	4	SGD	$1.0\times 10^{-2}$	$5.0\times 10^{-4}$	Poly	—
SegNeXt-L	MSCAN-L	2	AdamW	$6.0\times 10^{-5}$	$1.0\times 10^{-2}$	Lin. + Poly	Head lr_mult = 10
Mask2Former	Swin-L	1	AdamW	$1.0\times 10^{-4}$	0.05	Poly	Grad. clip (0.01)
